# Prognostic Significance of Preoperative Fibrinogen-to-Prealbumin Ratio in Patients with Stage I–III Colorectal Cancer Undergoing Surgical Resection: A Retrospective Cohort Study

**DOI:** 10.1155/2021/3905353

**Published:** 2021-01-12

**Authors:** Hailun Xie, Shizhen Huang, Guanghui Yuan, Shuangyi Tang, Jialiang Gan

**Affiliations:** ^1^Department of Colorectal and Anal Surgery, The First Affiliated Hospital, Guangxi Medical University, Nanning 530021, China; ^2^Department of Pharmacy, The First Affiliated Hospital, Guangxi Medical University, Nanning 530021, China

## Abstract

**Background:**

The objective of this study was to explore the role of preoperative fibrinogen-to-prealbumin ratio (FPR) in evaluating the prognosis of patients with stage I–III colorectal cancer (CRC).

**Methods:**

This retrospective study enrolled 584 stage I–III CRC patients undergoing surgical resection. Logistic regression analysis was used to explore the correlation between FPR and postoperative complications. The Kaplan-Meier curve and Cox proportional hazards model were used to identify the prognostic factors. The nomograms were constructed based on the prognostic factors. The concordance index and calibration curve were used to determine the accuracy of the nomograms. Time-dependent receiver operating characteristic was used to compare the predictive prognostic efficacy of nomograms and TNM stage.

**Results:**

FPR was determined to be an independent factor affecting postoperative complications. Patients with a low-FPR had a significantly better prognosis than those with a high-FPR (disease-free survival, *p* = 0.028; overall survival, *p* = 0.027), especially patients with stage I CRC (disease-free survival, *p* = 0.015; overall survival, *p* = 0.017). The Cox proportional hazards model identified FPR as an independent poor prognostic factor of disease-free survival (hazard ratio (HR) = 1.459, 95% confidence interval (CI) = 1.074–1.954, *p* = 0.011) and overall survival (HR = 1.405, 95% CI = 1.034–1.909, *p* = 0.030). The prognostic nomograms had good accuracy and were superior to the traditional TNM stage.

**Conclusions:**

FPR is a potential indicator for predicting short- and long-term prognosis of stage I–III CRC patients undergoing surgical resection.

## 1. Introduction

Colorectal cancer (CRC) is currently the third most common malignancy in the world, with 1.8 million new cases diagnosed every year. It is also the second leading cause of cancer-related deaths worldwide, accounting for approximately one-tenth of the total number of deaths [1]. Surgical resection of the primary tumor and subsequent adjuvant chemotherapy are still the main treatment strategies for CRC patients. With the advancement of surgical and adjuvant therapy techniques, the five-year overall survival (OS) in CRC patients has improved. However, a large proportion of patients undergoing primary tumor resection still die from tumor recurrence or metastasis [2, 3]. Hence, novel, effective, and economical prognostic biomarkers are urgently needed for prognosis evaluation and formulating individualized treatment strategies in CRC patients.

Inflammation and nutritional status are well known to play important roles in cancer patient survival. Tumor-promoting inflammation is a hallmark of cancer. An increasing number of studies have demonstrated that inflammation is closely related to carcinogenesis and progression of CRC [4, 5] and is one of the most critical reasons for the occurrence and metastasis of CRC [6]. In addition, Friis et al. have found that long-term use of low-dose non-steroidal anti-inflammatory drugs can reduce susceptibility to CRC [7]. Nutritional status is another important factor affecting the prognosis of CRC patients. Malnutrition can lead to undesirable clinical consequences, such as reduced body immunity, decreased therapeutic efficacy, and postoperative complications [8].

It has been recently reported that fibrinogen-to-prealbumin ratio (FPR) can be utilized to predict the prognosis of various malignancies, such as hepatocellular carcinoma, gastric cancer, and esophageal cancer [9–13]. Fibrinogen can promote the synthesis of proinflammatory cytokines and fibroblast growth factors, induce malignant tumor cell proliferation, promote tumor angiogenesis, and participate in cell responses related to tumor cell adhesion and migration [14, 15]. Several studies have demonstrated that preoperative plasma fibrinogen levels can independently predict the prognosis of various malignancies [16–18] including CRC. Prealbumin is an essential nutritional indicator with a short half-life that is considered to be more sensitive than albumin in reflecting protein energy status changes. It is also an effective biomarker that timely reflects inflammatory stress and tumor progression [19, 20]. Many studies have reported that low preoperative prealbumin level is associated with poor prognosis of various malignancies [21, 22]. Thus, FPR combined with fibrinogen and prealbumin can more reliably reflect the inflammatory and nutritional status of the host and is a potentially promising prognostic indicator.

At present, few researchers have reported the prognostic value of FPR in CRC patients, and no studies have explored the correlation between FPR and postoperative complications in CRC patients. Therefore, the present study retrospectively analyzed patient data from our center to explore the value of FPR in short- and long-term outcomes of stage I–III CRC patients undergoing surgical resection.

## 2. Materials and Methods

### 2.1. Patient Selection

A total of 584 patients with pathologically confirmed stage I–III CRC who underwent surgical resection at the First Affiliated Hospital of Guangxi Medical University between January 2012 and December 2014 were retrospectively analyzed. All enrolled patients underwent radical resection without preoperative neoadjuvant chemoradiotherapy. The exclusion criteria included patients presenting with systemic infection, hematological diseases, and additional malignancies, as well as individuals with incomplete preoperative serological data or those lost during postoperative follow-up. The research was approved by the institutional Ethics Committee of our center. Written informed consent was obtained from each patient.

### 2.2. Clinical Parameters and Laboratory Results

All enrolled patients were measured for serum neutrophil count, monocyte count, lymphocyte count, platelet count, fibrinogen, albumin, prealbumin, and carcinoembryonic antigen (CEA) levels within one week before surgery. Clinical data were collected using the hospital information system and included time of admission, length of hospitalization, gender, age, body mass index (BMI), preoperative comorbidities (diabetes, hypertension, and cardiovascular disease), postoperative chemotherapy, tumor location, type of surgery, depth of invasion, perineural invasion, vascular invasion, pathological type, histological grade, tumor size, blood loss, and operation duration. The comorbidities were classified according to the American Society of Anesthesiologists (ASA) classification. Tumor stages were classified according to the 8th tumor-node-metastasis (TNM) classification system of CRC. Prognostic study indicators were as follows: FPR = fibrinogen-to-prealbumin ratio, NLR = neutrophil-to-lymphocyte ratio, LMR = lymphocyte-to-monocyte ratio, PLR = platelet-to-lymphocyte ratio, and prognostic nutritional index (PNI) = albumin (g/L) + 5 × lymphocyte (10^9^/L).

### 2.3. Survival and Follow-Up

Follow-up data were collected by telephone interviews and retrieving outpatient and inpatient medical records. All included patients were regularly followed up every 3–6 months during the first two years and then every six months until September 1, 2019. The follow-up data consisted of medical history, physical examination, tumor marker detection, tumor assessment imaging, and colonoscopy. The clinical outcomes included postoperative complications, disease-free survival (DFS), and OS. Postoperative complications were defined using Clavien-Dindo (CD) classification of grade II or higher [23]. DFS was calculated from the date of operation until recurrence, metastasis, death, or the last follow-up. OS was calculated from the date of operation until death or last follow-up.

### 2.4. Statistical Analysis

The optimal threshold value for FPR was determined using X-tile software version 3.6.1 (Yale University School of Medicine, New Haven, CT, USA). The differences between groups were compared using a chi-square test, Fisher's exact test, or Student's **t**-test as appropriate. Univariate/multivariate logistic regression analysis was performed to investigate the relationship between FPR and postoperative complications. The Kaplan-Meier curve and log-rank test were used to assess the differences in survival rate. Survival analysis was assessed using the Cox proportional hazards model to determine potential prognostic factors. Factors with **p** values < 0.05 in univariate analysis were used for multivariate analysis. R software (Version 3.5.3; https://www.R-project.org) was used to establish the survival nomograms. Then, the performance of prognostic nomograms was evaluated using the concordance index (**C**-index) and calibration curve. Furthermore, the prognostic efficacy of survival nomograms and TNM stage was compared using time-dependent receiver operating characteristic (ROC) curve. SPSS 24.0 (IBM Corporation, Armonk, NY, USA) was used for all statistical analyses, and a **p** value < 0.05 with bilateral probability was considered statistically significant.

## 3. Results

### 3.1. General Study Population Information

CRC patient characteristics included in this study are summarized in Table 1. There were 363 males (62.2%) and 221 females (37.8%) with a median age of 59 years (range 17–92 years). Postoperative pathological results showed that the majority of patients had deep invasion (T3–4; 71.9%) and no lymph node metastasis (58.4%). There were 125 (21.4%) patients with stage I CRC, 215 with stage II (36.8%), and 244 with stage III (41.8%). Moreover, 215 patients (36.8%) had high preoperative CEA levels, and a large proportion of patients received postoperative adjuvant chemotherapy (50.7%). The median values for PNI, NLR, LMR, and PLR were 46.13, 2.14, 3.60, and 137.41, respectively, which were used as the thresholds. Using the OS values, X-tile software determined the optimal FPR threshold to be 23.1. Based on this threshold value, 357 patients had a low FPR and 227 patients had a high FPR.

The correlation between FPR and clinicopathological factors is analyzed in Table 1. FPR was significantly positively correlated with age (*p* = 0.002), tumor size (*p* = 0.002), CEA (*p* = 0.002), fibrinogen (*p* = 0.002), PNI (*p* < 0.001), NLR (*p* < 0.001), and PLR (*p* < 0.001). It was also significantly inversely correlated with BMI (*p* < 0.001), albumin (*p* < 0.001), prealbumin (*p* < 0.001), and LMR (*p* < 0.001). In addition, patients with colon and vascular invasion were more likely to have high FPR.

### 3.2. Correlation of FPR with Postoperative Complications

To accurately evaluate the correlation between FPR and postoperative complications, ≥grade II complications that required intervention were evaluated. Patients with a high FPR were more likely to have postoperative complications than those with a low FPR (16.3% vs. 9.0%, *p* < 0.007). To further determine the risk factors affecting postoperative complications, logistic regression analysis was performed based on various clinicopathologic factors. Age, hypertension, FPR, and blood loss were related to postoperative complications based on univariate analysis. However, multivariate analysis revealed that only high FPR (odd ratio (OR) = 1.785, 95% confidence interval (CI) = 1.062–2.999, *p* = 0.024) and blood loss (≥100 mL; OR = 1.956, 95% CI = 1.151–3.326, *p* = 0.013) were independent risk factors for postoperative complications (Table 2).

### 3.3. Comparison of Survival Curves between Low and High FPR

**Figure 1:**
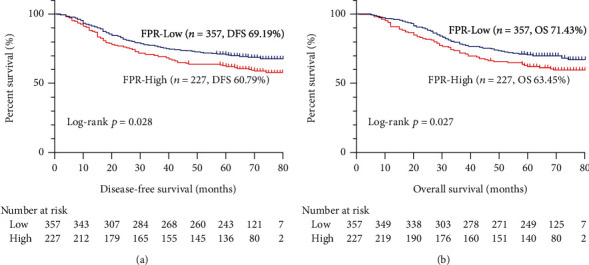
Kaplan-Meier curves of FPR in stage I-III CRC patients. Notes: (a) disease-free survival and (b) overall survival.

**Figure 2:**
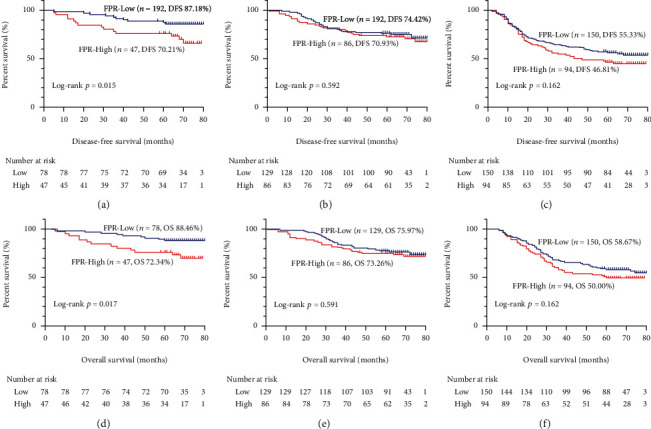
Stratified Kaplan-Meier curves analysis of FPR based on TNM stage. Notes: (a) disease-free survival of FPR in stage I CRC patients; (b) disease-free survival of FPR in stage II CRC patients; (c) disease-free survival of FPR in stage III CRC patients; (d) overall survival of FPR in stage I CRC patients; (e) overall survival of FPR in stage II CRC patients; (f) overall survival of FPR in stage III CRC patients.

The median follow-up period was 65 months (range 1–80 months). A total of 185 patients (31.68%) died, and 140 (23.97%) had confirmed recurrence or distant metastasis upon the last follow-up. The survival curve between low and high FPR was compared using the Kaplan-Meier method with log-rank test. The results revealed that patients with a high FPR had a significantly worse DFS compared with patients with a low FPR (*p* = 0.028). Their corresponding five-year survival rates were 60.79% and 69.19% (Figure 1(a)). Similarly, patients with a high FPR had a dramatically worse OS compared with patients with a low FPR (*p* = 0.027) with corresponding five-year survival rates of 63.45% and 71.43%, respectively (Figure 1(b)). The survival curves were further compared in each pathological stage. In stage I (Figures 2(a) and 2(d)), patients with a high FPR had a considerably worse DFS (70.21 vs. 87.18%, *p* = 0.015) and OS (72.34% vs. 88.46%, *p* = 0.017) than patients with a low FPR. In stages II (Figures 2(b) and 2(e)) and III (Figures 2(c) and 2(f)), the survival of patients with a high FPR was worse than that of patients with a low FPR, although these results were not statistically significant.

### 3.4. Correlation of FPR with Patient Survival

A variety of clinicopathological factors were incorporated into the Cox proportional hazards model for survival analysis. The univariate analysis showed that the patients with deep invasion, lymph node metastasis, perineural invasion, vascular invasion, low histological grade, high CEA, advanced ASA grade, and high FPR had adverse DFS. However, in the multivariable analysis, only lymph node status (N1 vs. N0, hazard ratio (HR) = 1.378, 95% CI = 0.958–1.983; N2 vs. N0, HR = 3.922, 95% CI = 2.590–5.938; *p* < 0.001), CEA (HR = 1.507, 95% CI = 1.133–2.004, *p* = 0.005), and FPR (HR = 1.459, 95% CI = 1.074–1.954, *p* = 0.011) were the independent prognostic factors of DFS (Table 3). Similarly, age, depth of invasion, lymph node status, vascular invasion, histological grade, CEA, ASA grade, and FPR were statistically associated with OS in univariate analysis. However, in multivariate analysis, only lymph node metastasis (N1 vs. N0, HR = 1.434, 95% CI = 0.978–2.103; N2 vs. N0, HR = 4.309, 95% CI = 2.797–6.638; *p* < 0.001), high CEA (HR = 1.400, 95% CI = 1.038–1.890, *p* = 0.028), and high FPR (HR = 1.405, 95% CI = 1.034–1.909, *p* = 0.038) were independent risk factors for OS (Table 4).

### 3.5. Establishing Prognostic Nomograms

**Figure 3:**
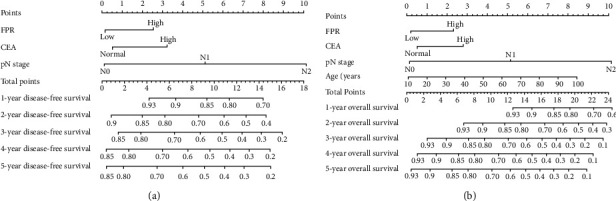
Construction of prognostic nomograms with FPR in stage I-III CRC patients for predicting 1-5-year DFS (a) and OS (b) in stage I-III CRC patients.

**Figure 4:**
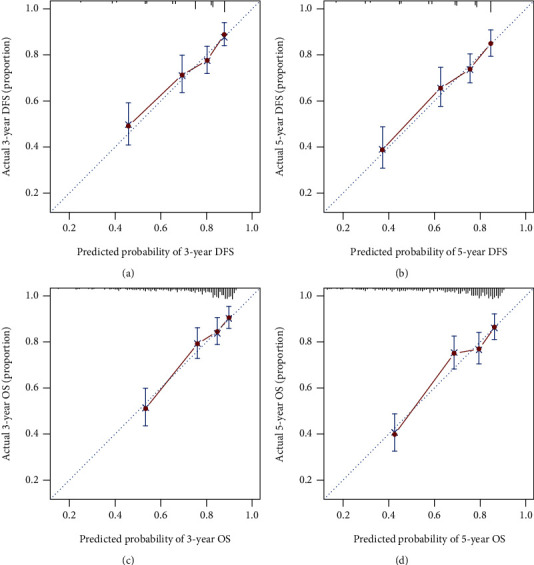
The calibration curves for predicting 3-year DFS (a), 5-year DFS (b), 3-year OS (c), and 5-year OS (d) in stage I-III CRC patients.

**Figure 5:**
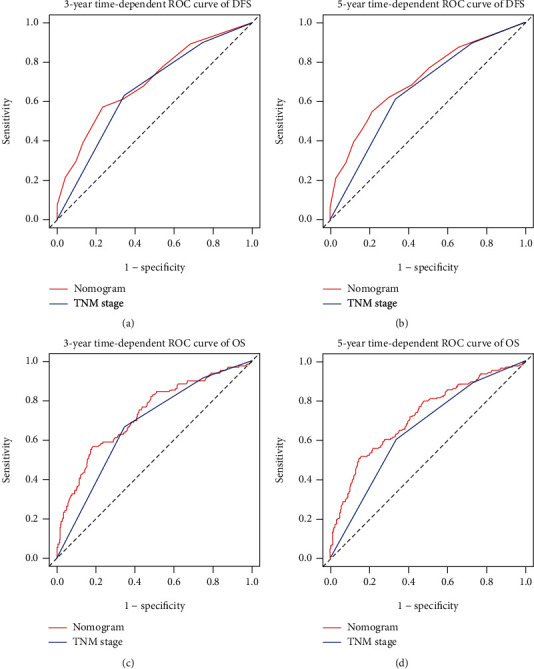
Comparison of the ability of prognostic nomograms and TNM stage for predicting prognosis in stage I-III CRC patients at 3-year and 5-year point. Notes: (a) DFS at 3-year point; (b) DFS at 5-year point; (c) OS at 3-year point; (d) OS at 5-year point.

Two simple and efficient prognostic nomograms were established for DFS (Figure 3(a)) and OS (Figure 3(b)) to predict the survival of stage I–III CRC patients undergoing surgical resection. The prognostic nomograms incorporated independent prognostic factors identified by multivariate analysis. The 1–5-year DFS and OS in CRC patients were estimated by calculating the sum of the scores for each factor. The *C*-indices of the prognostic nomograms for DFS and OS were 0.678 (0.641–0.715) and 0.689 (0.650–0.728), respectively. In addition, the calibration plot for DFS (Figures 4(a) and 4(b)) and OS (Figures 4(c) and 4(d)) at postoperative three- and five-year follow-up demonstrated an optimal agreement between the predicted prognostic nomograms and actual observations. The TNM staging system is widely recognized as the most effective scoring system to evaluate the prognosis in CRC patients. The predictive ability between the prognostic nomograms and TNM staging system was further compared using time-dependent ROC. The area under the ROC curve for the prognostic nomograms was larger than that for the TNM stage at the three-year survival point (Figures 5(a) and 5(c)) as follows: DFS—0.700, 95% CI = 0.651–0.750; TNM stage—0.656, 95% CI = 0.611–0.702 and OS, 0.729, 95% CI = 0.678–0.780; and TNM stage—0.674, 95% CI = 0.627–0.720. The area under the ROC curve for the prognostic nomograms was also larger than that for the TNM stage at the five-year survival point (Figures 5(b) and 5(d)) as follows: DFS—0.710, 95% CI = 0.664–0.756 and TNM stage—0.657, 95%CI = 0.613–0.701; OS—0.718, 95% CI = 0.671–0.765 and TNM stage, 0.649, 95% CI = 0.604–0.694.

## 4. Discussion

Systemic inflammation is an important hallmark of CRC [4]. Systemic inflammation can change tumor microenvironment, thereby promoting tumorigenesis and increasing proliferation, migration, and tumor cell immune escape [6, 24]. Systemic inflammation is usually determined using biochemical blood routine indicators, which have been reported in many studies to be associated with CRC progression and prognosis [25–27]. Nutritional status is another important factor affecting cancer patient prognosis. A gradual nutritional status decline can suppress tumor immunity, leading to tumor spread and disease progression [28, 29]. Our previous studies have found that perioperative malnutrition may increase the incidence of postoperative complications and delay the recovery of postoperative function, leading to prolonged hospitalization, reduced life treatment, and even poor survival outcomes [26, 30]. Various prognostic indicators based on systemic inflammatory and nutritional status have shown good performance in predicting the clinical outcomes in CRC patients, including Glasgow outcome score [31], modified Glasgow outcome score [32], and C-reactive protein/albumin [33].

The present study investigated the correlation between FPR and clinicopathological factors, complications, and survival in stage I–III CRC patients undergoing surgical resection. Correlation analysis results showed that high FPR was associated with low BMI, advanced age, and colon tumors, indicating that an increase in FPR may reflect the poor physical condition and nutritional status of patients. In addition, FPR is also correlated with tumor size and vascular invasion, suggesting that this biological indicator may be related to the aggressive cancer phenotype, which is consistent with previous studies [9, 34]. The relationship between FPR and complications was also assessed for the first time, revealing that patients with a high FPR had a higher incidence of complications, while high FPR was an independent risk factor for complications. In addition, survival analysis confirmed that FPR is an independent prognostic factor affecting both DFS and OS in CRC patients. In the stratified survival curve analysis, the survival rate for the low FPR group was significantly higher than that for the high FPR group in stage I. There were no statistical differences in stage II and III CRC, suggesting that FPR may be used for prognosis assessment in early stage CRC patients. This result may be explained by a greater influence of inflammatory nutrition-related factors on early tumor development compared to other factors. Tumor invasion and metastasis gradually acquire a dominant role as the tumor progresses, while the impact of inflammatory nutrition-related factors is weakened. Two prognostic nomograms were constructed to individually predict clinical outcomes, and both the *C*-index and calibration plot confirmed that these nomograms were a reliable risk stratification tool. The nomograms combine the pathological characteristics of the tumor itself, inflammatory nutritional biomarkers, and tumor markers and can provide a personalized prognostic risk assessment for each patient. The ability of these nomograms to predict CRC patient prognosis was compared to the TNM stage. The nomograms were superior to the TNM stage in predicting three- and five-year survival in CRC patients, which showed that they have certain advantages in individually predicting CRC patient prognosis and can be used to develop personalized postoperative follow-up and treatment strategies for CRC patients.

The reasons why FPR is related to CRC patient prognosis remain unclear. However, there are several possible mechanisms. Fibrinogen is a potential factor affecting the activity and biological behavior of tumor cells [15]. Palumbo et al. have found that lymphatic metastasis is reduced in mice with fibrinogen deficiency and suggested that fibrinogen is an important factor that maintains continuous adhesion and survival of tumor cells in the vasculature of target organs and promotes metastasis [35]. Zheng et al. have reported that fibrinogen and platelets promote mutual aggregation around tumor cells to form a platelet-fibrinogen protective film, thereby protecting tumor cells from natural killer cells [36]. Prealbumin is not only a commonly used nutritional marker but also an acute phase negative protein, which is negatively correlated with inflammation. Zhou et al. have reported that prealbumin, which has a shorter half-life, can be affected by acute changes in protein balance earlier and respond to nutritional needs faster than albumin, making it an effective predictor of short-term efficacy after gastrectomy [37]. Shen et al. have found that prealbumin can be used in combination with lymphocytes to predict long-term prognosis of gastric cancer [38]. Albumin is another important indicator for the assessment of nutrition and inflammation. However, albumin, prealbumin, and fibrinogen alone were not factors affecting postoperative complications and long-term prognosis of CRC patients in the present study. Because FPR balances the effects of inflammation and nutrition, it can more comprehensively reflect the biological status of patients. Patients with a high FPR represent an upregulation of fibrinogen and a decrease in prealbumin, which indicate that patients have a poor nutritional status and cancer-related inflammation burden. Chen et al. have suggested that high FPR represents significant severe chronic inflammation and malnutrition that increase chemotherapy resistance, while circulating FPR can help predict the progression and survival of patients with left-sided metastatic CRC [39]. Sun et al. have reported that FPR combined with tumor markers can assist in the identification of patients requiring adjuvant chemotherapy and help predict CRC patient prognosis [40].

The present study was the first to investigate the prognostic value of FPR in stage I–III CRC patients undergoing surgical resection. The results confirmed that FPR upregulation is related to the invasion biology and malnutrition of CRC patients. They also demonstrated that FPR is an independent predictor of short- and long-term prognosis in CRC patients. Notably, this study has some limitations. This was a single-center retrospective study with potential bias in patient selection and medical record inaccuracy. In addition, although the prognostic nomograms have proven to be superior to TNM staging in the present study, further large-scale studies from multiple centers are needed to verify these results in the future.

## 5. Conclusions

The present study confirmed that FPR is a potential indicator for predicting short- and long-term prognosis in stage I–III CRC patients undergoing surgical resection.

## Figures and Tables

**Table 1:** The correlations between the FPR and clinicopathological factors.

Variable	Case (584)	FPR		*χ* ^2^/*t*	*p*
		Low (357)	High (227)		
Sex				0.796	0.372
Male	363 (62.2%)	227 (63.6%)	136 (59.9%)		
Female	221 (37.8%)	130 (36.4%)	91 (40.1%)		
Age (years)	57.57 ± 13.41	56.31 ± 12.76	59.56 ± 14.16	2.877	0.004
BMI	22.11 ± 3.42	22.49 ± 3.35	21.51 ± 3.44	-3.385	0.001
Diabetes mellitus				3.513	0.061
Negative	553 (94.7%)	343 (96.1%)	210 (92.5%)		
Positive	31 (5.3%)	14 (3.9%)	17 (7.5%)		
Hypertension				2.114	0.146
Negative	492 (84.2%)	307 (86.0%)	185 (81.5%)		
Positive	92 (15.8%)	50 (14.0%)	42 (18.5%)		
Cardiovascular disease				0.096	0.757
Negative	570 (97.6%)	349 (97.8%)	221 (97.4%)		
Positive	14 (2.4%)	8 (2.2%)	6 (2.6%)		
Depth of invasion				1.178	0.278
T1-2	164 (28.1%)	106 (29.7%)	58 (25.6%)		
T3-4	420 (71.9%)	251 (70.3%)	169 (74.4%)		
Lymph node status				4.197	0.123
N0	341 (58.4%)	208 (58.3%)	133 (58.6%)		
N1	162 (27.7%)	92 (25.8%)	70 (30.8%)		
N2	81 (13.9%)	57 (16.0%)	24 (10.6%)		
Tumor stage				0.213	0.899
I	125 (21.4%)	78 (21.8%)	47 (20.7%)		
II	215 (36.8%)	129 (36.1%)	86 (37.9%)		
III	244 (41.8%)	150 (42.0%)	94 (41.4%)		
Tumor location				21.511	<0.001
Rectal	317 (54.3%)	221 (61.9%)	96 (42.3%)		
Colon	267 (45.7%)	136 (38.1%)	131 (57.7%)		
Perineural invasion				0.125	0.724
Negative	533 (91.3%)	327 (91.6%)	206 (90.7%)		
Positive	51 (8.7%)	30 (8.4%)	21 (9.3%)		
Vascular invasion					
Negative	501 (85.8%)	316 (88.5%)	185 (81.5%)	5.605	0.018
Positive	83 (14.2%)	41 (11.5%)	42 (18.5%)		
Pathological type				2.139	0.343
Protrude type	117 (20.0%)	68 (19.0%)	49 (21.6%)		
Infiltrating type	69 (11.8%)	38 (10.6%)	31 (13.7%)		
Ulcerative type	398 (68.2%)	251 (70.3%)	147 (64.8%)		
Histological grade				0.834	0.361
Low	61 (10.4%)	34 (9.5%)	27 (11.9%)		
High	523 (89.6%)	323 (90.5%)	200 (88.1%)		
Tumor size	4.93 ± 2.01	4.36 ± 1.60	5.83 ± 2.25	8.591	<0.001
ASA classification				0.291	0.590
I-II	322 (55.1%)	200 (56.0%)	122 (53.7%)		
III-IV	262 (44.9%)	157 (44.0%)	105 (46.3%)		
CEA				6.451	0.011
Normal	369 (63.2%)	240 (67.2%)	129 (56.8%)		
High	215 (36.8%)	117 (32.8%)	98 (43.2%)		
Length of hospitalization	14.44 ± 6.98	14.15 ± 6.33	14.89 ± 7.88	1.248	0.213
Albumin	36.90 ± 4.00	38.33 ± 3.13	34.64 ± 4.18	-12.164	<0.001
Prealbumin	203.88 ± 60.65	235.08 ± 47.42	154.80 ± 44.60	-20.686	<0.001
Fibrinogen	4.29 ± 1.03	3.82 ± 0.72	5.04 ± 0.98	17.364	<0.001
PNI	46.26 ± 6.38	48.12 ± 4.85	43.33 ± 7.34	8.689	<0.001
NLR	2.72 ± 2.36	2.26 ± 1.70	3.44 ± 3.00	5.389	<0.001
LMR	3.85 ± 1.93	4.20 ± 1.70	3.30 ± 2.13	-6.776	<0.001
PLR	158.76 ± 87.24	138.71 ± 71.83	190.29 ± 99.35	30.271	<0.001
Postoperative complications (CD ≥ 2)	69 (11.8%)	32 (9.0%)	37 (16.3%)	7.167	0.007

**Table 2:** The correlations between the postoperative complications and various clinicopathological factors.

Variable	Postoperative complication				
	Univariate		Multivariate		
	HR (95% CI)	*p* value	HR (95% CI)	*p* value	*β*
Gender (female)	0.801 (0.471-1.361)	0.412			
Age (years) (≥60)	1.675 (1.002-2.799)	0.049	1.311 (0.756-2.273)	0.335	0.271
BMI		0.876			
Low	1				
Normal	1.071 (0.514-2.232)				
High	0.916 (0.400-2.100)				
Diabetes mellitus (positive)	1.867 (0.737-4.726)	0.188			
Hypertension (positive)	2.103 (1.165-3.798)	0.014	1.843 (0.984-3.450)	0.056	0.611
Cardiovascular disease (positive)	1.251 (0.274-5.712)	0.772			
Depth of invasion (T3-4)	1.031 (0.588-1.809)	0.914			
Lymph node status		0.987			
N0	1.000				
N1	1.000 (0.559-1.788)				
N2	1.060 (0.506-2.221)				
Tumor location (colon)	0.844 (0.507-1.403)	0.513			
Perineural invasion (positive)	1.209 (0.522-2.801)	0.659			
Vascular invasion (positive)	1.476 (0.767-2.838)	0.243			
Pathological type		0.693			
Protrude type	1.000				
Infiltrating type	0.701 (0.256-1.917)				
Ulcerative type	1.033 (0.548-1.946)				
Histological grade (low)	1.764 (0.869-3.579)	0.116			
Tumor size (≥5 cm)	1.614 (0.968-2.689)	0.066			
CEA (high)	0.972 (0.576-1.639)	0.915			
ASA grade (III-IV)	1.072 (0.647-1.774)	0.788			
Type of surgery (open)	1.041 (0.630-1.722)	0.874			
Albumin (low)	1.278 (0.743-2.196)	0.375			
Prealbumin (low)	1.200 (0.704-2.044)	0.502			
Fibrinogen (high)	1.233 (0.883-1.720)	0.219			
FPR (high)	1.978 (1.193-3.280)	0.008	1.785 (1.062-2.999)	0.029	0.579
PNI (low)	1.540 (0.924-2.566)	0.097			
NLR (high)	1.104 (0.667-1.825)	0.701			
LMR (high)	0.649 (0.390-1.082)	0.097			
PLR (high)	1.440 (0.866-2.393)	0.160			
Blood loss (≥100 mL)	2.034 (1.204-3.438)	0.008	1.956 (1.151-3.326)	0.013	0.671
Operation time (≥209 min)	1.540 (0.924-2.566)	0.097			

**Table 3:** The correlations between the disease-free survival and various clinicopathological factors.

Variable	Disease-free survival			
	Univariate		Multivariate	
	HR (95% CI)	*p* value	HR (95% CI)	*p* value
Gender (female)	0.873 (0.652-1.168)	0.360		
Age (years) (≥60)	1.255 (0.949-1.659)	0.111		
BMI		0.736		
Low	1			
Normal	1.120 (0.742-1.689)			
High	0.999 (0.629-1.587)			
Diabetes mellitus (positive)	1.132 (0.631-2.029)	0.678		
Hypertension (positive)	1.229 (0.860-1.756)	0.258		
Cardiovascular disease (positive)	1.242 (0.461-3.342)	0.668		
Depth of invasion (T3-4)	1.951 (1.365-2.789)	<0.001	1.320 (0.907-1.922)	0.147
Lymph node status		<0.001		<0.001
N0	1.000		1.000	
N1	1.661 (1.190-2.320)		1.378 (0.958-1.983)	
N2	4.771 (3.399-6.697)		3.922 (2.590-5.938)	
Tumor location (colon)	0.852 (0.643-1.129)	0.266		
Perineural invasion (positive)	1.688 (1.109-2.567)	0.014	1.192 (0.748-1.899)	0.460
Vascular invasion (positive)	1.882 (1.338-2.646)	<0.001	1.188 (0.805-1.753)	0.385
Pathological type		0.335		
Protrude type	1.000			
Infiltrating type	1.285 (0.760-2.175)			
Ulcerative type	1.330 (0.911-1.943)			
Histological grade (low)	1.799 (1.226-2.640)	0.003	1.310 (0.883-1.943)	0.179
Maximum tumor size (≥5)	1.234 (0.934-1.630)	0.140		
CEA (high)	1.738 (1.315-2.296)	<0.001	1.507 (1.133-2.004)	0.005
ASA grade (III-IV)	1.919 (1.449-2.542)	<0.001	1.139 (0.822-1.578)	0.435
Type of surgery (open)	0.906 (0.686-1.197)	0.486		
Postoperative chemotherapy (yes)	1.019 (0.772-1.345)	0.896		
Albumin (low)	1.112 (0.781-1.584)	0.555		
Prealbumin (low)	1.051 (0.745-1.481)	0.778		
Fibrinogen (high)	1.163 (0.841-1.610)	0.362		
FPR (high)	1.365 (1.032-1.806)	0.029	1.459 (1.074-1.954)	0.011
PNI (low)	1.003 (0.760-1.325)	0.982		
NLR (high)	1.061 (0.803-1.401)	0.677		
LMR (high)	0.869 (0.658-1.148)	0.324		
PLR (high)	1.003 (0.760-1.325)	0.982		
Blood loss (≥100 mL)	1.110 (0.840-1.466)	0.464		
Operation time (≥209 min)	1.224 (0.926-1.618)	0.155		

**Table 4:** The correlations between the overall survival and various clinicopathological factors.

Variable	Overall survival			
	Univariate		Multivariate	
	HR (95% CI)	*p* value	HR (95% CI)	*p* value
Gender (female)	0.843 (0.622-1.142)	0.270		
Age (years) (≥60)	1.407 (1.051-1.883)	0.022	1.529 (1.122-2.086)	0.007
BMI		0.765		
Low	1			
Normal	1.001 (0.629-1.592)			
High	1.004 (0.717-1.408)			
Diabetes mellitus (positive)	1.115 (0.571-2.179)	0.750		
Hypertension (positive)	1.216 (0.838-1.763)	0.304		
Cardiovascular disease (positive)	1.590 (0.508-4.975)	0.426		
Depth of invasion (T3-4)	2.008 (1.378-2.926)	<0.001	1.409 (0.947-2.098)	0.091
Lymph node status		<0.001		<0.001
N0	1.000		1.000	
N1	1.624 (1.145-2.302)		1.434 (0.978-2.103)	
N2	4.805 (3.389-6.813)		4.309 (2.797-6.638)	
Tumor location (colon)	0.815 (0.608-1.093)	0.172		
Perineural invasion (positive)	1.487 (0.944-2.343)	0.087		
Vascular invasion (positive)	1.751 (1.226-2.502)	0.002	1.112 (0.763-1.621)	0.581
Pathological type		0.329		
Protrude type	1.000			
Infiltrating type	1.413 (0.826-2.418)			
Ulcerative type	1.325 (0.891-1.971)			
Histological grade (low)	1.919 (1.298-2.838)	0.001	1.495 (0.994-2.248)	0.053
Maximum tumor size (≥5)	1.298 (0.972-1.733)	0.077		
CEA (high)	1.698 (1.272-2.267)	<0.001	1.400 (1.038-1.890)	0.028
ASA grade (III-IV)	1.990 (1.485-2.666)	<0.001	1.104 (0.783-1.558)	0.572
Type of surgery (open)	1.122 (0.841-1.497)	0.435		
Postoperative chemotherapy (yes)	1.003 (0.752-1.339)	0.981		
Albumin (low)	1.173 (0.808-1.701)	0.401		
Prealbumin (low)	1.067 (0.747-1.524)	0.723		
Fibrinogen (high)	1.233 (0.883-1.720)	0.219		
FPR (high)	1.383 (1.035-1.848)	0.028	1.405 (1.034-1.909)	0.030
PNI (low)	1.048 (0.785-1.398)	0.751		
NLR (high)	1.031 (0.773-1.376)	0.835		
LMR (high)	0.891 (0.668-1.189)	0.434		
PLR (high)	1.001 (0.750-1.334)	0.999		
Blood loss (≥100 mL)	1.155 (0.865-1.542)	0.327		
Operation time (≥209 min)	1.286 (0.962-1.718)	0.090		

## Data Availability

The datasets generated and analyzed during the current study are not publicly available, because the data came from the hospital's independent and closed digital medical record management system. But they are available from the corresponding author on reasonable request.
